# Association Between Advance Care Planning for Older Adults and Family Caregivers’ Sense of Security

**DOI:** 10.1093/geroni/igab046.1377

**Published:** 2021-12-17

**Authors:** Masumi Shinohara, Mariko Sakka, Asa Inagaki, Taisuke Yasaka, Chie Fukui, Maiko Noguchi-Watanabe, Ayumi Igarashi, Noriko Yamamoto-Mitani

**Affiliations:** 1 University of Tokyo, Bunkyo, Tokyo, Japan; 2 The University of Tokyo, Bunkyo, Tokyo, Japan

## Abstract

Family caregivers’ (FCs’) sense of security benefits older adults who receive homecare. Advance care planning (ACP) is reported to have positive effects on FC’s experience, but it might differ depending on FCs’ kin relationships with the older adults. We examined whether ACP for older adults in homecare settings is associated with FCs’ sense of security. Further, we assessed whether such an association depends on their status as spouses or as adult children. We conducted a secondary analysis of data from a prospective cohort study in Japan. The participants were older adults who used home-visit nursing services, their FCs, and the nurses who cared for them. The FCs were asked to answer a sense of security questionnaire, and nurses were asked whether ACP was conducted. Multivariate logistic regression analyses were performed to examine the association between ACP implementation and positive changes in the sense of security scores after three months. Data from 169 cases were analyzed. Of the FCs, 28.1% were men and 55.6% were spouses. ACP was performed in 53.8% of the cases. The results of the multivariate analyses showed an interactive effect between ACP implementation and FC kin relationships. For spouses, ACP was significantly associated with a positive change in their sense of security. For adult children, such an association was not found. ACP might have a positive effect on caregiving spouses’ sense of security. Adult child caregivers, who often have multiple responsibilities and have difficulties facing their parents’ physical decline, may need support, in addition to ACP.

